# Herbal medicine for sports: a review

**DOI:** 10.1186/s12970-018-0218-y

**Published:** 2018-03-15

**Authors:** Maha Sellami, Olfa Slimeni, Andrzej Pokrywka, Goran Kuvačić, Lawrence D Hayes, Mirjana Milic, Johnny Padulo

**Affiliations:** 1 Faculty of Kinesiology, University of Split, Teslina 6, 21000 Split, Croatia; 2 Tunisian Research Laboratory, Sport Performance Optimization, National Center of Medicine and Science in Sports, Tunis, Tunisia; 3 Laboratory of Biosurveillance of the Environment, Faculty of Sciences of Bizerte, University of Carthage, Zarzouna, Tunisia; 4 Faculty of Medicine and Health Sciences, University of Zielona Gora, Zielona Gora, Poland; 5 Active Ageing Research Group, Department of Medical and Sport Sciences, University of Cumbria, Bowerham Road, Lancaster, UK; 6 University eCampus, Novedrate, Italy

**Keywords:** Ergogenic aid, Alkaloids, Polyphenol, Medicinal plant, Physical activity

## Abstract

*The use of herbal medicinal products and supplements has increased* during last decades. At present, some herbs are used to enhance muscle strength and body mass. Emergent evidence suggests that the health benefits from plants are attributed to their bioactive compounds such as Polyphenols, Terpenoids, and Alkaloids which have several physiological effects on the human body. At times, manufacturers launch numerous products with banned ingredient inside with inappropriate amounts or fake supplement inducing harmful side effect. Unfortunately up to date, there is no guarantee that herbal supplements are safe for anyone to use and it has not helped to clear the confusion surrounding the herbal use in sport field especially. Hence, the purpose of this review is to provide guidance on the efficacy and side effect of most used plants in sport. We have identified plants according to the following categories: Ginseng, alkaloids, and other purported herbal ergogenics such as *Tribulus Terrestris*, Cordyceps Sinensis. We found that most herbal supplement effects are likely due to activation of the central nervous system via stimulation of catecholamines. Ginseng was used as an endurance performance enhancer, while alkaloids supplementation resulted in improvements in sprint and cycling intense exercises. Despite it is prohibited, small amount of ephedrine was usually used in combination with caffeine to enhance muscle strength in trained individuals. Some other alkaloids such as green tea extracts have been used to improve body mass and composition in athletes. Other herb (i.e. Rhodiola, Astragalus) help relieve muscle and joint pain, but results about their effects on exercise performance are missing.

## Background

Athletes’ use of herbal supplements has increased tremendously over the past decade. Herbal products are extract from seeds, gums, roots, leaves, bark, berries, or flowers, and contain numbers of phytochemicals such as carotenoids and polyphenols, including phenolic acids, alkaloids, flavonoids, glycosides, saponins, and lignans which though to provide health benefits [1]. The use of herbal products is regulated by the Food and Drug Administration (FDA) as a special category of foods and classified as “dietary supplement” according to the Dietary Supplement Health and Education Act (DSHEA) of 1994 [1]. Herbold et al. [2] showed that 17% of collegiate female athletes have used herbal supplements. In sport, most supplements from herbs or plants were used to enhance muscle growth and fat burning [3]. Different commercial products such as “SportPharm” which contains numerous herbals, counting “Thermadrene”, “MaHuang”, “Guarana”, “Caffeine”, “Purple Willow Bark”, “Cayenne”, “pepper” and “Ginger root”, are believed to increase mental vigilance, stimulate fat-burning metabolism, and improves muscle performance [3]. Herbal supplements are currently used by athletes and non athletes alike to improve endurance and strength performance [3], however number of them have not proven safe and effective under current FDA standards. Others herbal dietary and botanical supplements were excluded from this requirement because they present a source of production of drugs [4]. Those herbs need to be explored further in humans.

Plants have been shown to provide several essentials metabolites such as carbohydrates, lipids, and nucleic acids and numbers of secondary metabolites such as terpenoids, alkaloids, and phenolic compounds. These later are widely sought for their biological properties: anti-allergic, anti-atherogenic, anti-inflammatory, hepato-protective, antimicrobial, antiviral, antibacterial, anticarcinogenic, antithrombotic, cardioprotective, and vasodilatory [5]. These biological properties are mediated by their antioxidant characteristics and redox properties. In fact, they play an important role in oxidative damage stabilization by free radical neutralization, oxygen scavenging, or decomposition of peroxides [6]. In this context, several studies highlighted the role of herbal supplements in reducing exercise induced oxidative stress in athletes [6, 7]. For some of them, reducing oxidative stress will enhance muscle recovery and energy maintenance during intensive exercises [3, 8, 9]. Authors suggested also that some products such as Ginseng, caffeine, and ephedrine are rich of antioxidant components and therefore are the best candidate to enhance muscle performances. Other plants such as *Tribulus Terrestris*, *Ginkgo biloba*, Rhodiolarosea, Cordyceps Sinensis have demonstrated benefits on muscle growth and strength in active men [3, 8, 9], while others [10–13] have demonstrated no effect on muscle performances. Heterogeneous clinical outcomes observed in previous studies are coming from different factors such as type of the plant, the geographic location from which the plant was gathered, and the method of extraction used. In addition, most of previous research highlighted the efficacy of herbal supplements without giving information about probable risk or negative side effect in athletes [14]. Irrespective of marketing natural supplements which are to improve health and physical performance, it should also be kept in mind, that some plants may have in their composition doping substances as well as some products based on herbal extracts may be contaminated or adulterated by agents prohibited in sport. As such, their real effects on sport performance remain inconclusive overall. In this review, we have identified the most used plants as supplement in sports. We have divided these products into following categories: Ginseng, herbal sources of caffeine and ephedrine and other purported herbal ergogenic plants such as *Tribulus Terrestris*, *Ginkgo biloba*, and *Rhodiola Rosea*.

### Ginseng

Ginseng is one of the best popular herbal dietary supplements and is probably the most studied herb with regards to physical performance [9]. Ginseng consists of numerous species in the Araliaceae family. There are several species of ginseng such as Asian ginseng, Korean ginseng, Chinese ginseng (*Panax ginseng*), American ginseng, Canadian ginseng (*Panax quinquefolius*) and Siberian ginseng (*Eleutherococcus senticosus*). Numerous Asian countries, particularly China and Korea use ginseng in the dietary and medicinal domain, whilst the *Panax ginseng* preparations have been elaborated in human clinical trials [9] such as an anti-inflammatory, antioxidant, a stimulant of brain function, anabolic and an immunostimulant, and an endurance performance enhancer. The ginseng species contains numerous important compounds such as the vitamins (A, B, C and E), minerals (iron, magnesium, potassium and phosphorus), fibers, proteins, saponins and *Ginsenosides* the main active constituents in Panax herbs. This component has been shown to reduce mental stress, improve immune function, and stabilizes blood pressure [15].

Siberian ginseng contains unique steroidal saponins named *Eleutherosides* which appears to be structurally similar to *Panax ginsenosides* [16] and contains phenolics and polysaccharides. Panax have been demonstrated to have ergogenic effects [17]. Small amount ≤ 200 mg/day of *Panax ginseng* (root powder or root extract with standardized *Ginsenoside* content), allows greater improvements in cognitive performance and anaerobic performance in untrained young or older subjects [8, 9].

In addition, *Ginseng* has important antioxidant properties, which inhibits hydroxyl radical and lipid peroxidation and facilitates mitochondrial activity during exercise [18]. It is considered an adaptogen agent with *Ginsenosides*, *Eleutherosides* and *Ciwujianosides* thought to be responsible for the ergogenic effect of ginseng [3]. Moreover, the chronic use of *Ginseng* improved cardio respiratory function and lower blood lactate concentrations, in addition to improving physical performance [19]. *Ginseng* ergogenic effect has been related to physical condition (Table 1). In fact, Bahrke and Morgan [16] found higher performances in sedentary and active individual compared with trained groups. In addition, Talbott and Hughes [20] observed that *ginseng* had beneficial effects on the central nervous system (CNS), adrenal and sexual function, with anti-fatigue properties in moderately trained individual. Other studies reported that the ginseng improves alertness, and fatigue resistance through cortisol stimulation [21].

**Table 1:** Selected studies on the effects of ginseng in exercise and sports

Study	Population	Dose	Period	Results
Forgo and Kirchdofer [28]	30 elite young athletes	200 mg/day of standardized ginseng extract, 4% or 7% ginsenoside content	9 weeks	↑ Aerobic capacity ↓ Lactate production, heart rate
McNaughton et al. [29]	30 subjects (15 females, 15 males)	1 g/day Chinese ginseng, Siberian ginseng or placebo	6 weeks	↑Maximal oxygen uptake (VO_2max_) ↑pectoral and quadriceps strength
Van Schepdael [30]	3 female triathletes aged 24 to 36 years old	400 mg/day ginseng extract	20 weeks	↑↑ Running time
Kim et al. [19]	7 healthy male adults untrained	6 g of *Panax ginseng* extract or placebo (3 time per day)	8 weeks	↑Cardio respiratory function ↓Lactate ↑Physical performance
Liang et al. [31]	29 untrained adults (age 20 to 30 years old)	1.35 mg/day Panax ginseng or placebo	30 days	↓Endurance running time
Ooiet al. [10]	8 male cyclists	0.1 mg of Eurycoma per 100 ml of drink or placebo drink	During exercise	Ø Cycling endurance performance
Hamzah and Yusof [25]	14 healthy men	150 mg of Eurycoma	5 weeks	↑ Muscle strength
Muhamad et al. [11]	12 recreational male athletes (ages 23.3 ± 3.7 years old)	2 capsules per day containing 75 mg of Eurycoma or placebo 2 capsules	7 days before exercise trial 1 h before exercise trial	ØRunning distance ØPhysiological Reponses between trials
Ping et al. [12]	9 recreational runners (ages 25.4 ± 6.9 years old)	200 mg of Panax ginseng	1 h before the exercise test	ØEndurance running time
Engels and Wirth [13]	36 healthy men	200 and 400 mg/day Panax ginseng or placebo	8 weeks	ØSubmaximal and maximal exercise permormance

↑augment, ↓reduce, Ø no changes

New data suggested that *Eurycoma longifolia Jack* (Malaysian ginseng) or Tongkat Ali is among the popular herbs that used to enhance exercise and sports performance and to treat several diseases and health issues [22], this herbs (the extract of its roots) allows to increase for men their libido and treat sexual disorders such as erectile dysfunction. It is a plant species of the family *Simaroubaceae*, found in Malaysia, Indonesia, Thailand, Vietnam and Laos. *Eurycomalongifolia Jack* contains *Quassinoids* and the *Squalene* derivatives *Biphenylneolignans, Tirucallane-Type Triterpenes*, *Canthine-6-Oneand Beta-Carboline* alkaloids which possess anti-inflammatory, anti-malarial, anti-ulcer, anti-cancer [23] and anti-plasmodial properties [24]. However, published scientific data regarding the effects of this plant on exercise performance are missing. Only few study found that *Eurycoma* supplementation (extract: 150 mg/day for 5-week) may increase muscle strength [25], while other study suggested that herbal drink containing *Eurycoma* (0.1 mg per 100 ml of drink) improved running performance in cyclists [10].

Like most supplements, *ginseng* has side effects, some of which are important depending on the dose and one’s metabolism. The use of *ginseng* has been shown to cause diarrhea, insomnia, headaches, rapid heartbeat, blood pressure fluctuations, and can cause digestive disorders. Women may experience additional side effects, such as vaginal bleeding and breast tenderness. Most of these side effects are serious enough to warrant stopping taking *ginseng* in breast cancer patient. Ginseng can interfere with various medications, such as insulin, digoxin, anticoagulants, and monoamine oxidase inhibitors. Moreover, it can be contraindicated in patient with high blood pressure [26]. As such, *ginseng* has a major limitation in healthy population. Nocerino et al. [27] stated, *ginseng* should be avoided by the energetic, nervous, tense, hysteric, or schizophrenic individuals, and should not be taken in combination with other stimulants, drugs or during hormones treatment. Hence, further research is needed to clarify the effects of major compound of *ginseng* in humans.

### Alkaloids

#### Caffeine

Caffeine is a natural compound found in plant species growing in the Tropic or Sub-Tropic regions of the world. This compound decrease the risk of degenerative brain diseases caused by aging (cognitive decline, dementia) and allows reducing the risk of Parkinson’s disease. Caffeine is an alkaloid that may be perceived to be ergogenic. In fact, caffeine may offer greater benefits on both endurance and anaerobic performances [32, 33]. According to Kovacs et al. [34], a small quantity of caffeine (≈2 to 9 mg/kg body mass) taken at least 1-h prior to exercise or competition stimulates greater improvements in some measures of strength, and increases serum catecholamine levels and immune responses in runner and cyclist [35]. Higher blood catecholamines have been found to increase anaerobic performances (i.e., sprint performances) and aerobic performances (VO_2max_) in healthy young and middle-aged individuals [35, 36]. Caffeine supplementation can improve also performance at different exercise intensities levels [37] as well as mental vigilance and humor [8]. Graham and Spriet [38] observed a significant increase in endurance running performance after ingestion of 9 mg/kg body mass of caffeine 1-h prior to exercise. Collomp et al. [39] investigated the effects of caffeine ingestion on sprint performance in trained and untrained swimmers and reported subjects’ swimming velocity and maximal blood lactate concentration was significantly improved in both untrained and trained subjects after caffeine ingestion. Other study found that ingestion of 1-2 mg/kg caffeine at breakfast decreases reaction time during exercise and improves mental alertness [40]. Some evidence suggested that caffeine ergogenic effect is due to its antioxidant property [41] and its effect on free fatty acids (FFA) [42]. In fact, Ping et al. [43] found an increase in endurance performance and higher amount of plasma FFA following caffeine supplementation (5 mg/kg body weight).

Caffeine can have impressive health benefits, but high doses can also lead to negative side effects. In fact, it has been shown that excessive and chronic use of caffeine can lead to episodes of anxiety and high blood pressure [44]. High dose’s caffeine (> 400 mg/day) can cause anger one’s stomach lining, disrupt sleep, cause diarrhea and increase dehydration [45]. Despite those minor negative effects, the World Anti-Doping Agency (WADA) removed caffeine from the Prohibited List [46] and its use in sport is still monitored. There are also several plants considered as herbal sources of caffeine commonly found in supplement products and include “*Coffea Arabica*”, “*Guarana*” (*Paulliniacupana*), “*Kola nut*” (*Cola Acuminate*), “*Green tea*” (*Camilla sinensis*) and “*Mate*” (*Ilex Paraguayensis*) [9].

#### *Coffea Arabica*

*Coffea Arabica* is a species of Coffea originally indigenous to the forests of the south-western highlands of peninsula in Northeast Africa. Coffea may have similar effects to caffeine’s, as coffea is a complex mixture resulting from a hot-water extract of roasted coffee beans. Although many biological mechanisms are attributed to caffeine’s action as an adenosine antagonist which increases many neurotransmitter activities [32]. Rafiul Haque et al. [47] found that *Coffea arabica* seeds have stimulatory effect on cellular immune function in mice.

#### Guarana (*Paullinia cupana*)

Guarana, also known as *Guarana Gum*, *Guarana Seed*, *Zoom Cocoa* and *Brazilian Cocoa*, is native herbal from Amazon region. The active compounds in *Guarana* are the alkaloids: *Caffeine*, *Theophylline*, *Theobromine*, *Tannins* and *Saponins*. According to Natural Medicines Database [48], Guarana contains higher amount of caffeine than most plants with 3.6% to 5.8% of caffeine compared to 1% to 2% in coffea. Guarana has been found to activate central nervous system (i.e., increase of mental vigilance, fatigue resistance) improve body weight [49]. The seeds from this South American jungle shrub are regularly used to treat headaches, paralysis, urinary tract irritation, and diarrhea. Memorial Sloan-Kettering [26] found that Guarana is thought to interact with many types of supplements and medicaments such as products that contains caffeine, monoamine oxidase inhibitors, adenosine, clozapine, lithium, and acetaminophen. In fact, Boozer et al. [50] found that the addition of 72 mg of Ephedra and 240 mg caffeine from Guarana for 8-week reduced body mass and fat in active individual. Guarana may have serious side effects for some indivuduals. The appetite suppressant effect is related to the caffeine content. In general, the side effects reported from guarana use are related to its caffeine content and include anxiety, insomnia, rapid heart beat and upset stomach.

#### Green tea

Green tea (*Camilla Sinensis*) extract is one of the important herbal supplements that have recently been used to prevent weight gain [51] and stimulate nervous system [52]. It contains higher amounts of caffeine as well as *Catechin Polyphenols*, *Theobromine* and *Theophylline* which possess antioxidant properties and increase energy expenditure by stimulating brown adipose tissue thermogenesis [52]. In fact, Dullo et al. [51] found that combination of green tea with caffeine (50 mg caffeine and 90 mg of *Epigallo catechin Gallate* for 3 times per day) increased significantly 24-h energy expenditure and fat utilization in active individuals.

Green tea extracts (GTE) supplementation has been found to increase endurance capacity, improve the antioxidant defense system, and muscle lipid oxidation in healthy or diabetic individuals [53]. In addition, it increases plasma glycerol and epinephrine levels following sprint training in trained and untrained men [54]. Furthermore, supplementation of GTE reduces oxidative DNA damage induced by exercise after 14-day in untrained obese men [55] and after 4 weeks in sprinters [56]. Howevr, Jówko et al. [56] reported no changes in antioxidant enzyme or sprint performances after GTE supplementation in sprinters.

Interestingly, there is no published data on the effects of long term GTE supplementation on antioxidant biomarkers, plasma muscle damage parameters in trained individual and most studies using GTE supplementation did not assess the amount of other active components in green tea which would underestimate or overestimate the role of GTE on oxidative stress balance.

With regard to its harmful effects, it has been shown that epigallocatechin-3-gallate contained in green tea may induce greater cytotoxicity to liver cells and a higher amount (> 5% of diet for 13 weeks) can prompt oxidative DNA damage of pancreas cells with alterations in thyroid function [57].

#### Theobromine and theophylline

*Theobromine* and *Theophylline* are found in many plants like kola and tea. In sport, athletes used the *Chocolate* and *Cocoa* form as a principal source of these alkaloids [35]. In this last review, authors reported higher antioxidant status with no immune function alterations following *Cocoa* ingestion in healthy trained and untrained individuals. Till today, there are few studies that investigate the effect of definite doses of these alkaloids in athlete but the results are inconclusive, mostly because of incomplete data related to its compounds.

#### Mate

*Mate* (*Ilex paraguayensis*) or *Yerba mate* is a small evergreen holly tree that cultivates in various countries of South America. The tea made from the dried leaves contains about 2% caffeine [9]. In recent years, it has been suggested that the caffeine found in mate, kola nut, and guarana is more likely to benefit health than caffeine found in coffee or tea [58]. *Yerba mate* supplementation decreased body fat mass, body fat, and waist-hip ratio in obese individuals without significant negative [59]. Hoffman et al. [60] found significant increase in energy expenditure in young and healthy individuals after ingestion of supplement containing 317 mg of *Yerba Mate*. However, ingestion of this supplement resulted in higher heart rate and systolic blood pressure and confusion among subjects [60]. As such uses of this extract must be taken with precaution and more research are needed to fix the safe amount to be used in humans.

### Ephedrine

Ephedrine is an alkaloid with ergogenic properties that can be found in plants of the Ephedra type. The Ephedrine is a potent pharmacologic agent with various peripheral and central effects. Numerous studies have found a link between Ephedrine ingestion and higher physical performances and [3, 61] and weight loss [62]. In fact, Ephedrine has been used as a medical drug and a stimulant to treat low blood pressure, urinary incontinence, narcolepsy and depression [63]. It is currently used as a treatment of bronchial asthma, nasal inflammation, and the common cold [64]. It also enhances aerobic capacity, reduces fatigue, increases alertness, and reaction time during exercise [65].

However, Ephedrine uses was usually combined with caffeine in major studies which limit his potential role compared to caffeine [3, 9]. In fact, in these previous studies, different dose of caffeine (≤300 mg) and Ephedrine (≤70 mg) were used in recreational, runners and resistance trained athletes and showed decrease in running time and increase in muscle performances. Molnár et al. [66] found that combination of ephedrine and caffeine (oral ingestion) improve weight loss in adolescents, with mild and temporary negative side effects. In some other few studies, when Ephedrine was taken alone, there was no improvement in physical performance [67]. It should be emphasized that both ephedrine and its derivatives (*Cathine, Methylephedrine, Pseudoephedrine*) are considered to be doping substances, and higher doses would allow several harmful effect on body’s health [68, 69]. They are prohibited by the World Anti-Doping Agency (WADA) [46] in sports competition. Uses of ephedrine have been linked also to sleeping disorder, anxiety, headache, hallucinations, high blood pressure, fast heart rate, loss of appetite, and inability to urinate in several cases [70, 71].

### Ginger

Ginger is found in the tropical rainforest in Southern Asia and it includes alkaloids. Ginger (*Zingiberofficinale Roscoe*; *Zingiberaceae*) is a flowering plant that has been used in medicine for decades. Ginger has few negative side effects and it is listed in the FDA’s “safe” list [71]. Ginger has been shown to have anti-inflammatory effect in vitro studies [35] which is may be due to *Gingerols*, *Paradols*, *Shogaols*, their congeners, or other compounds. Till today, few studies have demonstrated analgesic effect of Ginger and fatigue resistance in athletes, while few other studies have not found any effect on body composition, metabolic rate, oxygen consumption and muscle strength in athletes [72].

Nakhostin-Roohi et al. [73] explored the effect of 150 mg curcumin (*Curcuma longa*) supplementation immediately after intensive eccentric squat exercise in healthy young males. They found decreased levels of serum inflammation biomarkers (creatine kinase (CK), alanine aminotransferase (ALT), and aspartate aminotransferase (AST)) in experimental group compared with placebo group. Curcumin is a diaryl heptanoid, belonging to the group of curcuminoids and a member of the ginger family (*Zingiberaceae*) and composed mainly by phenols. About harmful effect, it was demonstrated that high doses of curcumin (> 8 g/day for 3–4 months) had no toxic effect in most cancer patients, while few number of patients had nausea or diarrhea [74].

Various clinical experiments have demonstrated the safety and efficacy of oral curcumin supplements in various health conditions and most results have been positive for a significant proportion with no or minimal toxicity. However, adverse effects were all related to higher doses and should be avoided in in pregnancy and lactation. In addition, due to its anti-platelet property, it is recommended to avoid higher doses in patient with bleeding disorders.

### Other plant with ergogenic properties

#### *Tribulus Terrestris*

Extracts of *Tribulus Terrestris* (TT), a flowering plant distributed in the world, have been used to treat urinary tract infections, urolithiasis, dysmenorrhea, edema, hypertension, and hypercholesterolemia [3]. The most important chemical compositions of this plant are steroids; *Saponins* like *Dioscine, Diosgenin*, and the *Protodioscin*. These elements can have beneficial effects on libido and physical fitness. It also contains *Phytosterols*, in particular *Beta-Sistosterols*, which is beneficial for the prostate function, the urinary system and the cardiovascular system. In sports, plant gained wide recognition when medal-winning Bulgarian athletes from the 1996 Summer Olympics in Atlanta gave credit to TT for their success. Recent scientific studies found that *Tribulus Terrestris* extract (TTE) improves testosterone production in healthy male [75, 76]. Ivanova et al. [75] found that well-trained athletes and weightlifters used TTE supplementation to enhance production of luteinizing hormone (LH) and muscle growth. By increasing testosterone, reducing inflammation and oxidative damage in muscle, TT appears to be a potent performance enhancer [76]. TT is considered to be a safe alternative for the treatment of several diseases such as cardiovascular and Hypoactive sexual desire disorder (HSDD) in postmenopausal women [76]. Other studies have shown that TT supplementation (3.2 mg/kg body mass) has no effects on body composition and maximal strength (5-weeks: 450 mg of a TTE) [77], muscular endurance in resistance-trained men [7], testosterone levels in response to short period (5 day: 750 mg/day) or moderate long period (4-weeks: 10 or 20 mg/kg body mass) [78] [79] in trained individuals.

Nevertheless, there are still athletes who use TT to enhance their sports performance, athletes who are mainly coming from strength and power sports (e.g., weight lifting, sprint, throwing disciplines). This could be explained by the intensive marketing, which may only result in a temporary placebo effect caused by TT supplementation [14]. Despite the beneficial effect of TT supplementation on muscle performance, this plant could lead to a positive doping control test (Australian Institute of Sport [80]; the National Centre for Sports Medicine in Poland and the Medical Commission of the Polish Olympic Committee [81]; Canadian Cycling Association [82]). Although it is thought to be relatively safe, higher dose of TT (≥1000 mg per day) could lead to sleeping disorder, burnout and fatigue, hypertension, and high heart rate [83, 84]. Hence, uses of TT should be taken with precaution to avoid negative health issue.

### *Rhodiola Rosea*

*Rhodiola Rosea* (RR) is a popular herb used in traditional medicine in Europe and Asia. RR, also known as the *Pink Orpine*, or *Lignum Rhodium* belongs to the *Crassulaceae* family. It is found in Scandinavia, Central Asia or the Arctic, France especially in the Pyrenees and the Alps. The most used part in this plant is the root. RR is composed by *Rosine Rosarines*, *Rosin*, *Tyrosol*, *Rosiridin*, *Tannins* and *Polyphenols*. The most important compounds are *Salidroside* and *Rosavin* [85]. It contains also minerals, vitamins, gallic acid and chlorogenic acid as well as antioxidants that have an effective action to fight the aging of the skin. RR has been used to treat stress and anxiety syndrome, prevent high altitude sickness, and stimulate the nervous system. These benefits are due to natural components of the root that would activate the production of four molecules: norepinephrine, serotonin, dopamine and acetylcholine. These molecules act directly on the cerebral cortex and increase attention, memory, concentration and intellectual capacity, increase fatigue resistance, and physical performance [85].

In addition, recent data reported numerous benefits of RR characterized by antioxidant properties and adaptogenic effects especially for weakness syndrome [85]. Walpurgis et al. [86] reported that supplements of root or rhizome extracts of RR were found to contain significant amounts of the endogenous Steroids 4-androstene-3,17-dione and Dehydroepiandrosterone (DHEA) and the Pseudoendogenous Steroid 1,4-androstadiene-3,17-dione. However, there does not appear to be any reports documenting the occurrence of anabolic androgenic steroids.

Most of previous effects were attributed to the plant component such as phenolics (Salidrosides, p-Tyrosol, and Glycosides like Rosavins) which are considered as antioxidant element [85]. The study of DeBock et al. [87] found that the consumption of RR (200 mg/day) improved time to exhaustion by 3% on a cycle ergometer, but there was no significant effect following 4 weeks of supplementation and no effect on maximal strength or reaction time. Parisi et al. [88] found also that 4 weeks of RR supplementation can reduce lactate levels and muscle damage biomarkers in response to aerobic exercise in trained athletes. In addition, 3 mg/kg of RR ingestion has ability to decrease heart rate response to submaximal exercise and to lower the perception of effort during high-intensity endurance exercise in recreationally fit women [89].

The combination of RR with other plants extracts has shown no ergogenic effect on oxygen consumption, cycling time or muscle strength [56, 90]. Similarly, Earnest et al. [91] have found no effect on oxygen uptake and muscle performance during maximal graded test following 14 days of RR supplementation (300 mg) with Cordyceps (800 mg). According to Ahmed et al. [92], RR ingestion did not enhance immune system response of marathon athletes.

At present there is no evidence or mechanism to explain positive effect of this herb in sport, hence further researchers are needed.

Due to divergent data, Food and Drug Administration (FDA) reported that this herb does not present sufficient safety to be declared as new drug or safe supplement and therefore listed it on the FDA’s Poisonous Plant Database.

### Codyceps Sinensis

Natural *CordycepsSinensis* (CS) is an entomopathogenic fungus found in Asia mountain region. It is an ascomycete fungus that belongs to the family of Clavicipitaceae and to the order of the Hypocreales. It is has been demonstrated efficient role for the treatment of cholesterol and immune system disorder. The available synthetic version is CordyMax Cs-4. Chemical composition includes amino acids, Stearic Acid, D-Mannitol, Mycose, Ergosterol, Uracil, Adrenine, Adenosine Palmitic Acid, Cholesterol Palmitate and 5alpha-8alpha-epidioxy-5alpha-ergosta-6,22-dien-3beta-ol. It has been used to improve renal function in patients with chronic allograft nephropathy [93] and regulate blood pressure by stimulating vessel dilation, activating the nitric oxide production, and increasing the oxygen exchanges through capillary barrier [94]. CS was found to induce higher endurance performance [95]. Li et al. [96] found increase in hemoglobin levels following CS supplementation (5 days of 100–150 mg/kg) with greater aerobic capacity. CS extracts supplementation (powder form) appear to increase muscle fatigue resistance by enhancing lactic acid production, heart rate variability and blood pressure during maximal graded test in sedentary subjects [97]. In addition, a 240 mg of *Cordyceps* drink has been shown to improve cardiovascular responses in healthy runners [98].

However, recent research has shown that oral CS supplementation for 8-weeks have no effect on steroids hormones in resistance-trained adults [99]. Parcell et al. [100] reported that 5 weeks of CordyMax Cs-4 supplementation (3 g/day) had no effect on aerobic capacity or endurance performance in well-trained cyclists. Even longer period of CS supplementation (8 weeks: 2.4 g/day) had no significant ergogenic effect and did not affect testosterone level of resistance-trained athletes [99]. But, it appears that the combination of CS with other plants extracts like Rhodiola crenulata have great benefits on aerobic performance in well trained athletes [101]. Despite some benefits, there is insufficient evidence about the role of CS supplementation in athletic performance.

When taken in high doses, CS can cause stomach trouble and diarrhea. Hence, it is important to fix the safe dose and duration for human before beginning to consider it as an ergogenic aid.

### *Ginkgo biloba*

*Ginkgo biloba* (GB) is one of the most popular herbs growing in Asia. The active compounds for this herbal are the Flavonoids and Terpenoids [102]. Their content of flavonoids promotes the blood circulation and in particular cerebral blood circulation; it is therefore used in disorders that seem to be due to a reduction in blood flow such as Alzheimer’s disease, memory loss, migraines and headaches. It is also used in the treatment of Parkinson’s disease. In fact, GB has been found to activate the release of endothelium-derived relaxing factor, which may enhance muscle tissue blood flow through improved microcirculation [103].

The leaf of *Ginkgo biloba* presents high concentration of flavonoids which allow important antioxidant capacity. As such, the modern medicine uses leaf extracts from GB to create natural commercial product (i.e., EGb 761®, Tanakan® or Tebonin®). However, some products such as EGb 761 are not yet approved by FDA in U.S, but still available only by prescription in Europe.

In sport, Schneider [104] found that GB enhances endurance performance (longer walking distance) in patients with peripheral arterial disease (PAD). However, Wang et al. [105] showed that GB supplementation (24 weeks: 2 × 120 mg/day tablets) has no effect on walking economy or walking performance in patients with PAD. Zhang et al. [106] found that 7-week of GB ingestion combined with RR improve the endurance performance (higher VO_2max_) and time to exhaustion in healthy young athletes. Recently, numerous researches observed amelioration in cognitive performance especially in elderly with dementia syndrome [107, 108].

Despite its beneficial effects, Ginkgo was considered as safe herb only when taken by healthy adults by mouth and at limited doses. Ginkgo appears also to reduce blood glucose, hence, it important to take precaution in people with diabetes or hypoglycemia or following anaerobic exercises [109].

### Cayenne

Cayenne (*Capsicum Frutescens, Capsicum Annuum*) is considered as most commonly used spices. The Capsicum species are grown in tropical America in the Solanacées family. The active compound for this species is capsaicin [110] and its pain-relieving action is related from its capacity to interfere with sensory nerve signaling in the skin [26]. Cayenne has been used to treat diarrhea, cramps, and muscle inflammation [111]. Mason et al. [112] suggested that only one of every eight patients treated with 0.025% capsaicin attain 50% reduction in pain. Acute capsaicin supplementation has been shown to enhance resistance training performance (i.e. total weight lifted), with significantly higher blood lactate in trained group compared to placebo [113]. Lim et al. [114] found that the use of capsaicin (10 g of dried, hot red pepper) induce sympathetic nervous stimulation and increase lipid oxidation in long distance runners. In mice, Capsaicin supplementation decreases muscle soreness and increases muscle strength [115]. In the United States Pharmacopeia, the capsaicin is classified as a stimulant and its effects are similar to physiological action of caffeine. Although cayenne has many benefits, it can cause unpleasant reactions such as itching, burning sensation on the skin but these signs disappear fast. With its limited side effects, cayenne appears to be safe and useful to treat muscular related fatigue and overtraining syndrome.

### Arnica

Arnica (*Arnica Montana*) is a perennial herb in the Asteraceae family, grown in southern Russia, Europe, and America. The active compounds of Arnica are Flavonoids, Thymol, Arnicin, Coumarins, and Carotenoids. It was used to reduce the risk of cardiovascular events in patients with clinically stable coronary disease, to treat acute and chronic inflammation, infectious diseases and to stimulate the immune function [116]. Short term (3 to 6-week) uses of Arnica help reduce pain and increase muscle strength in patient with osteoarthritis of the knee [117]. Some studies suggested that the use of Arnica decreases muscle soreness and cell damage after marathon in well trained athletes [118, 119], while other study did not observe any change in physical performance following arnica supplementation [120]. On the other hand, the Arnica consumption at high doses has been shown to cause severe skin irritations [121], and oral consumption which can cause fatal poisonings in high amounts [122].

### Astragalus

*Astragalus* (*Astragalusmembranaceus*) is a perennial herb in the Fabaceae family. The active compounds for this plant are Saponins and Polysaccharides which have important effect on immune system such as natural killer cells activity [123]. In addition, it increases white cell count and levels of interferon in patient immunosuppression [124]. Most studies on effect of *Astragalus* have been conducted in cancer patient and reported a potential role in the treatment of inflammation [125].

In sport, Chen et al. [126] found that 8-week of *Astragalus* supplementation increased aerobic performance in runner compared to placebo groups. Rogers et al. [127] found out that Ginseng and *Astragalus* (1% flavonoids for the *Astragalus* fraction) may provide health and psychological benefits such as lowering cholesterol levels and improving self-reported levels of energy. However, there are no indications on standalone doses, duration, and methods of *Astragalus* supplementation which does not allow us to conclude about its real efficiency in humans. Due to the lack of report on severe side effects, *Astragalus* is believed to be safe with few side effects such as immune system suppression at higher doses [128].

### *Salix alba*

*Salix alba* (*White Willow*) is a tree of the *Salicaceae* family native to Europe and western and central Asia. It contains Salicin, which is converted to acetylsalicylic acid inside intestine. The willow bark has been used to treat pain, inflammation, osteoarthritis, aches and to reduce fevers [129]. In fact, short period of willow bark supplement (240 mg salicin/day for 2-week) decreases joint pain in patients with osteoarthritis [130], while longer period (6-week) does not seem to improve this symptom [131]. In addition, oral administration of 120 mg or 240 mg salicin for 4-week reduces back pain in patient with low back pain [132]. In sport, this extract has been used to treat musculoskeletal and joint-related conditions (injuries, inflammation) [133]. However, no studies were conducted to investigate the ergogenic effect of this herb on muscle performance in athletes, which present a limitation of his actual use in sports field. Shara and Stohs [129] suggested that adverse effects appear to be minor when compared to non-steroidal anti-inflammatory drugs such as aspirin.

### Herbs marketed with limited scientific research

Several other plants such as *Yohimbe, Spirulina (Arthrospiraplatensis and Arthrospira maxima), Moringa* (*Moringaoleifera*)*, Bala* (*Sidacordifolia*) *and Camu CamuMyrciariadubia*) have been used as source of proteins, minerals, and vitamins (VitB12 and Vit C). They were found to reduce body mass and increase endurance performance in runners and bodybuilders. Some of these plants have shown greater antioxidant capacity (i.e. *Myrciariadubia, Biostimine*, extract from *Aloe arborescens Mill*) [134].Other plants such as *Lignosus Rhinocerotis* [135], *Citrus aurantium* [136, 137] have been used in combination with exercise training or with caffeine to enhance performances of young athletes. They were also found to be effective in reducing muscle soreness but failed to demonstrate any improvements in anaerobic or aerobic performances. *Lignosus Rhinoceros* (medicinal mushroom) for example has been extensively used safely without specific side effect in human or animals, while extracts from *Citrus Aurantium* are believed to induce similar harmful side effect of *Ephedra* [138].

### Saffron (*Crocus sativus* Linn.)

Saffron is derived from the flower of *Crocus sativus* cultivated in Greece regions and its dried extract contains B vitamins, flavnoids and dietary minerals (mainly Magnesium, Calcium and Potassium). It contains several volatile and aroma-yielding compounds such as *Terpenes, Terpene Alcohol*, and their esters. *C. Sativus* have several beneficial effects such as antihypertensive, anticonvulsant, antitussive, antigenototoxic and cytotoxic effects, anxiolytic aphrodisiac, antioxidant, antidepressant, antinociceptive, anti-inflammatory, and relaxant activity. It has been shown to enhance memory and learning skills, and increases blood flow in choroid and retina.

In sport, Hosseinzadeh et al. [139] demonstrated that 4-week of saffron supplementation (30 mg/day) reduces levels of Lactate dehydrogenase (LDH), tumor necrosis factor alpha (TNF-α), and creatine kinase (CK) in sedentary women following one bout of acute resistance exercises at 85% of one-repetition maximum. In this study, no changes in resistance exercise performances were detected. In addition, 16-week of saffron supplementation (90 mg/day) has also been shown to reduce 8-Isoprostane levels and increased superoxide dismutase (SOD), catalase (CAT) activity in seminal plasma and sperm DNA damage in young healthy nonprofessional cyclists [140]. This plant and its extracts have demonstrated harmful effects at doses > 5 g per day and can causes death at 20 g per day [141], hence, it should be taken with precaution.

### Fenugreek (*Trigonella foenum-graecum*)

Other plant in the family of Fabaceae is believed to be safe and have also positive effect on glucose metabolism and digestion process in human, the *Fenugreek* [142, 143]. Reported data on the *Fenugreek* identified 32 phenolic compounds among which flavonoid glycosides and phenolic acid are detected [144]. Their seed contains alkaloids, coumarins, vitamins, and saponins [144]. In sport, Fenugreek extract has been demonstrated to improve endurance capacity and fatty acids utilization in male mice [145]. In human, Wankhede et al. [146] found that 8-week of Fenugreek supplementation (1 capsule of 300 mg, twice/day) showed beneficial effects on body fat, free testosterone levels, serum creatinine, but without change in kidney profile (enzymes) or side effects in male subjects during resistance training. Despite its safety, some people may develop or have an existing allergy to Fenugreek ingestion, some of these allergies are diarrhea, dyspepsia, abdominal distention, flatulence, hypoglycemia in diabetics persons [147].

### *Myrtus communis* L.

*Myrtus comminus* L. is a species found in the myrtaceae family, native to the Mediterranean basin. Many phenolic compounds were identified in *Myrtus communis L*. berry such us phenolic acids (gallic acid, caffeic acid, syringic acid, vanillic acid and ferulic acid), flavonoids (quercetin, myricetin) and hydrolyzable tannins (gallotannins). Myricetin and its glycoside derivatives are the major constituents of myrtle berries [148, 149]. Myrtle fruits are a high phenolic content, especially the anthocyanins for that it is among the fruits with the highest antioxidant activity [150, 151]. In addition Myrtle fruit was charaterized by higher levels of Linoleic Acid and low and varied proportions of saturated acids [149].

Recent studies have demonstrated the benefits of myrtle fruit (*Myrtus communis* L.) as a supplement in sport. In fact, Slimeni et al. [150] demonstrated that 4 weeks of myrtle fruit supplementation (3.4 mg/kg /day) may increase anaerobic performances, serum proteins and Iron and reduce triglycerides, in moderately trained athletes.

It have several properties such as antiseptic, astringent, carminative, hair tonic, analgesic, cardiotonic, diuretic, anti-inflammatory, stomachic, nephroprotective, antidote, brain tonic and antidiabetic [152], but presents minor harmful effects such as diharrea and nausea following ingestion of high doses (> 4 mg /day) [150].

## Conclusions

Today, many athletes have turned to various dietetic interventions, including the use of natural products based on herbs and plants to avoid risk from synthetic drug. However, it is imperative to have a comprehensive and extensive guide, which allows expert and athletes to understand beneficial and harmful effect of some product better. In this context, we have found that most herbs (Table 2) used in sports have a low-moderate effect on oxidative stress, fatigue resistance, and endurance capacity.

**Table 2:** Summarized table

Plants	Physical performance	Overall health
*Ginseng*	↑Aerobic capacity ↑Cardio respiratory function ↓Lactate ↑Physical performance ↓Endurance running time Ø Cycling endurance performance ↑ Muscle strength	Anti-inflammatory, antioxidant ↑brain function Immunostimulant ↑virility Treat digestive disorders ↓ mental stress ↑immune function Stabilizes blood pressure
*Caffeine* *Coffea Arabica* *Guarana* *Green Tea* *Theobromine* *Mate*	↑endurance running performance ↑muscle strength, ↑serum catecholamine levels ↑ immune responses in runner and cyclist ↑ blood catecholamine ↑anaerobic performances ↑endurance running performance ↑ endurance capacity	↓ The risk of degenerative brain diseases caused by aging (cognitive decline, dementia) ↓ the risk of parkinson’s disease ↑central nervous system (i.e., increase of mental vigilance, fatigue resistance) ↑ body composition ↑treat headaches, paralysis, urinary tract irritation, and diarrhea ↑blood pressure, anxiety, headaches, and cardiac stimulation ↑ the antioxidant defense system, and muscle lipid oxidation in healthy or diabetic individuals
*Ephedrine*	↑ aerobic capacity ↓fatigue ↑ alertness and reaction time	↑treat low blood pressure, urinary incontinence, narcolepsy and depression ↑for treatment of bronchial asthma, nasal inflammation, and the common cold
*Ginger*	↑fatigue resistance in athletes, Ø body composition, Ø oxygen consumption Ø muscle strength in athletes ↓inflammation biomarkers (creatine kinase (CK), alanine aminotransferase (ALT), and aspartate aminotransferase (AST)	anti-inflammatory Ø metabolic rate
*Tribulus Terrestris*	↑ testosterone production in healthy male ↑ production of luteinizing hormone (LH) ↑ Muscle growth. ↓inflammation ↓ oxidative damage in muscle, ↑cardiovascular and HSDD Ø body composition Ø maximal strength Ø muscular endurance in resistance-trained males during training	↓hypertension, and ↓hypercholesterolemia ↑ libido ↑ prostate, ↑ urinary system ↑ cardiovascular system
*Rhodiola Rosea*	↑ muscle fatigue resistance ↑ performance ↑ time to exhaustion by 3% on a cycle ergometer, Ø maximal strength reduce lactate levels Ø muscle damage parameters after cardio-pulmonary exhaustion test in trained male athletes Ø oxygen consumption, cycling time or muscle strength Ø oxygen uptake and muscle performance	↑digestive system, ↑cardiovascular system ↑sexual function and ↑libido. ↓stress and anxiety syndrome, ↑nervous system ↑norepinephrine, serotonin, dopamine and acetylcholine. ↑attention, memory, concentration and intellectual capacity, Ø immune system response of marathon athletes
*Cordyceps Sinensis*	↑aerobic capacity ↑muscle fatigue resistance ↑lactic acid production, heart rate variability and blood pressure during maximal graded test in sedentary subjects ↑cardiovascular responses in health runners Ø on steroids hormones in resistance-trained young male adults Ø on aerobic capacity or endurance exercise performance in endurance-trained male cyclists.	↑ treatment of cholesterol ↑ Immune system ↑ stimulation sexual ↓stress ↑ renal function in patients with chronic allograft nephropathy ↑regulate blood pressure by stimulating vessel dilation ↑ the nitric oxide production, ↑oxygen exchanges through capillary barrier
*Ginkgo biloba*	↑muscle tissue blood improved microcirculation enhances exercise performance Ø on improvement of walking economy in patients with PAD. ↑the endurance performance lending to greater VO_2max_ and time to exhaustion in health young.	↑circulation of blood and in particular cerebral blood circulation ↓Alzheimer’s disease, ↑memory ↓migraines and headaches. Parkinson’s disease ↑cognitive performance especially in elderly with dementia syndrome
*Cayenne*	↑ resistance training performance	↑ treat diarrhea, cramps, and muscle inflammation ↑ stimulation of sympathetic nervous and greater lipid oxidation in long distance male runners
*Arnica*	↓muscle soreness and cell damage after distance running in marathon runners ↑muscle strength in patient with osteoarthritis of the knee ↓pain	↓cardiovascular risk ↑treat inflammatory, infectious diseases ↑ immune system
*Astragalus*	↑ aerobic performance in runner	↑immune system ↑treatment for inflammation in cancer disease
*Salix alba*	↑to treat musculoskeletal and joint-related conditions (injuries, inflammation)	↑treat pain, inflammation, osteoarthritis, aches ↓fevers ↓joint pain in patients with osteoarthritis ↓ back pain in patient with low back pain
*Saffron*	↓levels of Lactate dehydrogenase (LDH), tumor necrosis factor alpha (TNF-α), and creatine kinase (CK) in sedentary women following one bout of acute resistance exercises ↑superoxide dismutase (SOD), catalase (CAT) activity in seminal plasma and sperm DNA damage in young healthy nonprofessional cyclists	Antihypertensive, anticonvulsant, antitussive, antigenototoxic and cytotoxic effects, anxiolytic aphrodisiac, antioxidant, antidepressant, antinociceptive, anti-inflammatory, and relaxant activity. ↑memory and learning skills ↑ blood flow in choroid and retina
*Fenugreek*	↑endurance capacity and fatty acids	↑free testosterone levels ↓serum creatinine Ø in kidney profile (enzymes)
*Myrtus Communis*	↑ anaerobic performances, serum proteins and Iron ↓triglycerides	antiseptic, astringent, carminative, hair tonic, analgesic, cardiotonic, diuretic, anti-inflammatory, stomachic, nephroprotective, antidote, brain tonic and antidiabetic

↑improve, ↓reduce, Ø no effect

Ginseng and caffeine had greater effect on central nervous system and appear to increase alertness and reaction time, while other herbs seem to stimulate steroids hormone production such as TT. Despite their positive effects, these herbs should be used with precaution because high doses may cause harmful side effects on kidney and stomach in particular.

## Abbreviations

ALT: Alanine aminotransferase
AST: Aspartate aminotransferase
CK: Creatine kinase
CNS: Central nervous system
CS: *Cordyceps Sinensis*
DSHEA: Dietary supplement health and education act
FDA: Food and drug administration
GB: *Ginkgo biloba*
GTE: Green tea extracts
LH: Luteinizing hormone
RR: *Rhodiola Rosea*
TT: *Tribulus Terrestris*
TTE: *Tribulus Terrestris* extract
WADA: World anti-doping agency
